# Biological effects of biochar and zeolite used for remediation of soil contaminated with toxic heavy metals

**DOI:** 10.1038/s41598-021-86446-1

**Published:** 2021-03-26

**Authors:** Tomasz Głąb, Krzysztof Gondek, Monika Mierzwa–Hersztek

**Affiliations:** 1 Department of Machinery Exploitation, Ergonomics and Production Processes, University of Agriculture in Krakow, ul. Balicka 116B, 31-149 Krakow, Poland; 2 Department of Agricultural and Environmental Chemistry, University of Agriculture in Krakow, Al. Mickiewicza 21, 31-120 Krakow, Poland; 3 Department of Mineralogy, Petrography and Geochemistry, AGH University of Science and Technology, Al. Mickiewicza 30, 30-059 Krakow, Poland

**Keywords:** Environmental impact, Agroecology, Grassland ecology

## Abstract

Biochar and zeolite are widely used in the remediation of soil contaminated with toxic heavy metals. However, the interaction of these amendments and their effects on grass productivity have not been comprehensively summarized. The aim of this study was to investigate the biological effects of zeolite and biochar used as soil amendments in the process of remediating soil contaminated with Cd, Pb and Zn. In a pot experiment, the following treatments were applied: zeolite, biochars produced at temperatures of 350 °C and 550 °C, mixtures of biochars and zeolite, and a control treatment without any amendments. The soil amendments were tested on two grass species: tall fescue (*Festuca arundinacea* Schreb.) and cocksfoot (*Dactylis glomerata* L.). The root morphometric parameters and aboveground production were determined in 2017 and 2018.

Higher biomass production was observed in the tested grasses in the treatments with zeolite alone (0.229 kg DM m^−2^) or mixed with the biochars (0.239 kg DM m^−2^) than in control treatment (0.029 kg DM m^−2^). Zeolite used in contaminated soil significantly affected root biomass and root morphology parameters. Zeolite application resulted in significantly higher root biomass (2.30 mg cm^−3^) and root length (76.61 cm cm^−3^) than those in the treatments without zeolite (0.29 mg cm^−3^ and 6.90 cm cm^−3^). Biochar as a soil amendment did not affect most root morphometric parameters. The application of biochars only slightly reduced the root diameter of cocksfoot. The root diameter of tall fescue was similar in all treatments (0.075 mm) except the control (0.063 mm) and biochar 550 treatments (0.067 mm), in which slightly thinner roots were observed.

## Introduction

Soil toxic metal contamination has become a serious concern in many regions worldwide^1,2^. According to a report by He et al.^3^, globally, more than 50% of areas with polluted soils are contaminated with heavy metals. Heavy metals in soil come from natural and anthropogenic sources. The main anthropogenic sources of toxic metals in soil are industrial processes, mining, domestic and municipal wastes, agrochemicals, and petrol-derived products^4^. Heavy metals are incorporated into food chains from polluted soil and cause forage and food contamination, thus affecting human and animal health. Thus, it has become a significant challenge to control and remove toxic metals from the environment.

To date, three reclamation techniques have been developed for metal-contaminated soils: biological, agricultural engineering, and physicochemical techniques^5^. The biological method of removing metals from contaminated soil, i.e., phytoremediation, is carried out through the planting, harvesting and removal of metal-accumulating crops^6^. Agricultural engineering methods consist of upper soil layer replacement or deep ploughing. Physicochemical immobilization is a process in which toxic metals are transferred into a chemical form that is insoluble or not active in biological systems^7^. Various soil amendments, e.g., lime, phosphate, kaolinite, bentonite, steel slag, red mud, biochar and zeolites, have been recognized to be able to ameliorate contaminated soils by immobilizing toxic metals^8,9^.

Zeolites are crystalline aluminium–silicate minerals with porous structures and high specific surface areas. They are found in natural volcanic rocks and can be synthesized from different Si and Al materials. Zeolites are characterized by their high capacity for adsorption, cation exchange and catalysis^5^. These traits make them widely used as soil amendments with possible applications for soil reclamation through toxic metal adsorption^10,11^. Lahori et al.^10^ reported a reduction in Pb, Cd, Cu and Zn contents in cabbage and corn roots and shoots after the application of zeolite. Research by Yi et al.^12^ showed that zeolite increased the soil pH and reduced the biological activity of heavy metals in the soil of a *Brassica juncea* L. crop.

Biochar has also been recognized as a soil amendment that can immobilize toxic metals^9,13^. It is a solid product of the thermal decomposition of biomass under oxygen-limited conditions. Biochars may vary in their chemical and physical properties. This variability depends on the parameters involved in the pyrolysis process and the materials used to produce the biochar^14^. During the past decade, biochar has been recognized as a soil amendment that provides opportunities for soil improvement and mitigates climate change by promoting carbon sequestration^15^. Biochar amendment has a beneficial influence on the physical, chemical, and biological properties of soil^16,17^. Biochar is a very porous material with a high surface area, similar to zeolite^18^. This porous structure and its chemical characteristics, such as its functional groups (e.g., carboxyl, hydroxyl, and phenolic), mean that biochar has a strong affinity for heavy metals^13^. Bashir et al.^9^ observed that biochar reduced Cd leaching by stabilizing Cd within the soil profile. Biochars prepared from chicken manure and green waste significantly reduced Cd, Cu and Pb accumulation in *Brassica juncea*^13^.

Using biochar and zeolite is considered an eco-friendly and cost-effective method of soil remediation^9^. However, there is a lack of up-to-date research reports concerning the interaction between biochar and zeolite in soil remediation for crop production. In particular, little is known about the impact of this remediation technique on perennial plants, e.g., grasses. Grasses are characterized by high tolerance to heavy metals and are therefore widely used in phytoremediation^19^.

The objective of this study was to determine the biological effects of zeolite and biochar application on soil contaminated with toxic metals. The effectiveness of this remediation technique was tested on the growth of two grass species, tall fescue and cocksfoot, with a special focus on their root morphometric parameters.

## Material and methods

### Site description and experimental design

A pot-based experiment was conducted at the University of Agriculture in Krakow, south of Poland (50°04′ N, 19°51′ E, 280 m a.s.l) in 2017–2018. The climate of the experimental site was temperate. The average annual temperature during the study period was 7.7 °C. The pots were placed in a rainfall shelter without walls and with a transparent glass roof to prevent precipitation but to ensure natural light and ventilation.

Loamy sand soil was contaminated with the following toxic metals: Cd (3CdSO_4_ ∙ 8H_2_O) at a rate of 2.50 mg kg^−1^, Pb (C_4_H_6_O_4_Pb ∙ 3H_2_O) at a rate of 300 mg kg^-1^ and Zn (ZnSO_4_ ∙ 7H_2_O) at a rate of 500 mg kg^−1^. Soil was thoroughly mixed with the water solution with toxic metals to ensure homogeneity then the pots (diameter of 0.22 m, volume of 0.009 m^−3^) were filled with contaminated soil.

The physical and chemical characteristics of the soil are presented in Table 1. Soil moisture was maintained at 55% of the maximum water holding capacity.

**Table 1:** Basic soil physical and chemical properties (means ± standard deviation).

Parameter	Unit	Value
pH (H_2_O)		5.67 ± 0.05
Solid particle density	g cm^−3^	2.65 ± 0.06
Sand	g kg^−1^	850
Silt	g kg^−1^	90
Clay	g kg^−1^	60
Soil organic carbon	g kg^−1^	6.43 ± 0.08
Total N	g kg^−1^	0.54 ± 0.01
P	mg kg^−1^	188 ± 11
K	mg kg^−1^	305 ± 29
Ca	mg kg^−1^	207 ± 17.9
Mg	mg kg^−1^	236 ± 12.5
EC	µS cm^−1^	32.2 ± 4.35
Cd	mg kg^−1^	0.19 ± 0.01
Pb	mg kg^−1^	37.9 ± 0.5
Zn	mg kg^−1^	16.5 ± 0.1

The experiment was conducted with a completely randomized design in three replications with two factors: (1) grass species and (2) soil amendment. The following treatments were applied: zeolite (Z), biochars (B350 and B550) and mixtures of biochars and zeolite (B350 + Z and B550 + Z). A control treatment (CTR) without any amendments was also established. Zeolite and biochar were applied at a rate of 0.5%, which corresponds to 15 t ha^−1^. After amendment application, two grass species, tall fescue (*Festuca arundinacea* Schreb.) and cocksfoot (*Dactylis glomerata* L.), were sown at a rate of 3.5 g per pot.

### Zeolite and biochar

**Figure 1:**
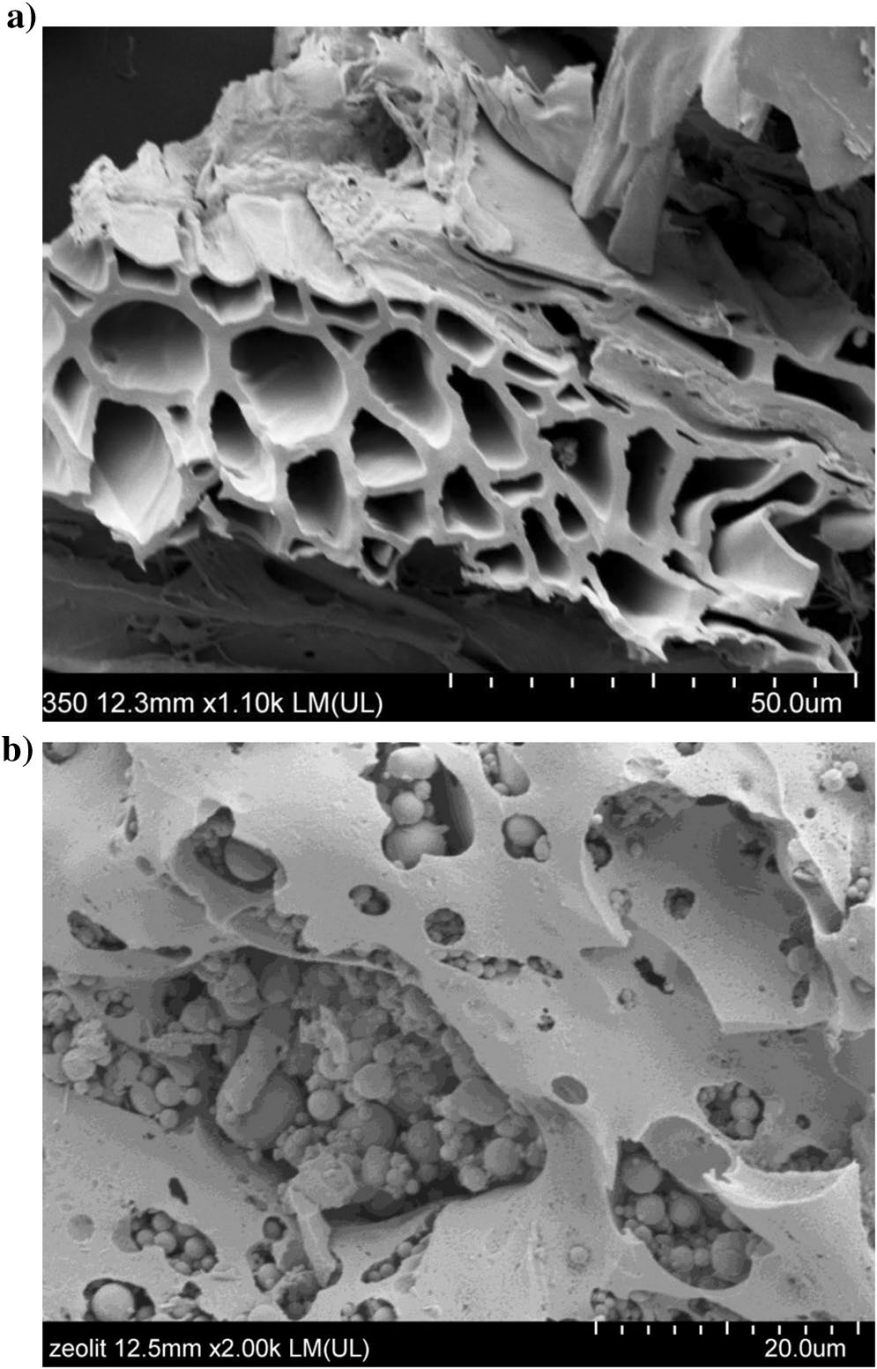
SEM images of (**a**) biochar produced from willow (*Salix* ssp.), (**b**) zeolite synthesized from coal fly ash with Na-P1 structure.

The zeolite was synthesized by a hydrothermal method from coal fly ash (Kozienice Power Plant, Poland). The ash sample was mixed with 0.5 dm^3^ 3 M NaOH and heated at 75 °C for 24 h. The zeolite was characterized by its Na-P1 structure^20^ (Fig. 1).

Biochar was produced from willow (*Salix *ssp.) biomass. The willow chips were air-dried at 70 °C, ground into particles with diameter below 4 mm, and mixed for homogeneity. Then, the feedstock was pyrolyzed in an electric furnace at a temperature of 350 °C for 15 min (B350) and at 550 °C for 15 min (B550)^21,22^. The furnace heating increased with a rate of 10 °C min^−1^. The basic chemical characteristics of the feedstocks are presented in Table 2. The specific surface area and porosity were determined from N_2_ gas adsorption/desorption isotherms at − 196 °C using an ASAP 2020 apparatus (Micromeritics, Norcross, GA, USA). Prior to measurements, the samples were outgassed for 12 h at 105 °C. Based on the data obtained from N2 isotherms, specific surface area (*S*_*Bet*_) was calculated by applying Brunauer–Emmett–Teller (BET) equation^23^. The total pore volume was calculated from the amount of N_2_ adsorbed at a relative vapor pressure (P/P0) ~ 0.99^24^. Biochar morphology is presented with SEM images as shown in Fig. 1.

**Table 2:** Chemical properties of amendments used in the experiment (means ± standard deviation).

Parameter	Unit	Biochar (B350)	Biochar (B550)	Zeolite (Z)
pH (H_2_O)		4.96 ± 0.16	8.34 ± 0.15	12.39 ± 0.05
EC*	mS cm^−1^	0.40 ± 0.01	0.65 ± 0.03	2.28 ± 0.11
DM**	g kg^−1^	980 ± 1	985 ± 0	997 ± 1
Total C	g kg^−1^ DM	691 ± 4	795 ± 11	3.43 ± 0.00
Total N	g kg^−1^ DM	5.15 ± 0.31	5.61 ± 0.08	0.48 ± 0.01
K	g kg^−1^ DM	3.88 ± 0.05	5.84 ± 0.04	3.22 ± 0.05
P	g kg^−1^ DM	0.44 ± 0.01	0.62 ± 0.02	1.62 ± 0.02
S	g·kg^−1^ DM	0.82 ± 0.13	0.91 ± 0.05	0.130 ± 0.003
Na	g·kg^−1^ DM	0.160 ± 0.001	0.220 ± 0.003	0.998 ± 0.011
Mg	g·kg^−1^ DM	0.89 ± 0.03	1.24 ± 0.02	3.63 ± 0.08
Ca	g·kg^−1^ DM	10.43 ± 0.14	14.56 ± 0.36	46.69 ± 1.91
Zn	mg·kg^−1^ DM	112.20 ± 2.29	155.53 ± 4.73	82.78 ± 2.64
Cd	mg·kg^−1^ DM	0.320 ± 0.02	1.65 ± 0.02	0.614 ± 0.027
Pb	mg·kg^−1^ DM	5.81 ± 0.30	14.44 ± 1.35	24.82 ± 0.28
Specific surface area (*S*_*Bet*_)	m^2^ g^−1^	1.36	2.01	74.91
Total pore volume	cm^3^ g^−1^	0.003	0.001	0.225

*EC-electrical conductivity; **DM-dry matter.

### Measurements

Roots were collected from pots in 2018 using the core method (diameter of 50 mm) with three replication per pot. The samples were prepared to analysis using a hydro-pneumatic washing system^25^ to separate soil particles from the roots. Digital images of root samples were obtained with an Epson Perfection 4870 photo scanner (Seiko Epson Corp., Owa Suwa, Japan) and saved as tiff at a resolution of 1200 dpi. The images analyse was performed using Aphelion 3.2 software (ADCIS S.A. and Amerinex Applied Imaging, Herouville, Saint-Clair, France). The images were analysed by method described by Bauhus and Messier^26^. The roots were divided into following diameter classes: < 0.02, 0.02–0.05, 0.05–0.1, 0.1–0.2, 0.2–0.5, 0.5–1.0, 1.0–2.0, and > 2.0 mm. The root morphometric parameters were calculated by methods described by Głąb et al.^27^ i.e.: root length density (RLD), mean root diameter (MRD, root surface area density (RSAD), specific root length (SRL) and root volume density (RVD). The roots were dried at a temperature of 70 °C to determine the root dry matter density (RDMD).

The grasses were harvested in June and September in 2017 and 2018. Harvesting was performed by cutting the plants 5 cm above the soil layer. Aboveground biomass samples were taken from each pot and dried for 48 h at 80 °C for the calculation of the dry matter yield.

### Statistics

The statistical analyses were performed with the Statistica v. 13.3 software package (StatSoft Inc., Tulsa, OK, USA). To avoid pseudoreplication, means for the replication per pot were used in ANOVA analyses. Two-way ANOVA was used to evaluate the significance of the soil amendments and grass species on biomass production and root morphology. Data normality was checked using Shapiro–Wilk test. The homogeneity of variance was checked using Levene’s test. Means comparison was analysed by Bonferroni test at the *P* < 0.05. Pearson's correlation and regression models were used to analyse the relationship between root morphological parameters and grass yields and between the below and above-ground biomass.

## Results

### Zeolite and biochar affect grass yields

**Figure 2:**
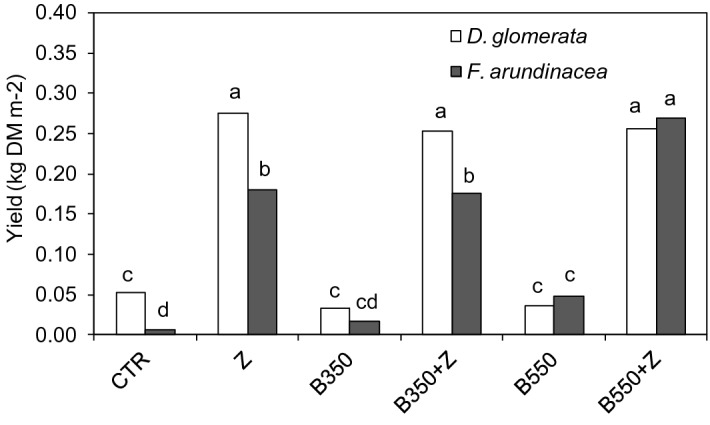
Yields of aboveground biomass of tall fescue (*F. arundinacea*) and cocksfoot (*D. glomerata)*. Treatments: zeolite (Z), biochars (B350 and B550), mixtures of biochars and zeolite (B350 + Z and B550 + Z), control (CTR). Different letters on the bars indicate significant differences by the Bonferroni test (*P* < 0.05, n = 3).

The highest aboveground biomass of tall fescue was obtained at the 2nd cut, with 0.090 and 0.064 kg DM m^−2^ in 2017 and 2018, respectively. The tall fescue yield obtained during the 1st cut was 0.039 kg DM m^−2^ on average (Table 3). A similar effect was also observed for the biomass production of cocksfoot. Cocksfoot had higher total annual productivity (0.151 kg DM m^−2^ on average) than tall fescue (0.116 kg DM m^−2^) (Fig. 2).

**Table 3:** Aboveground biomass production (kg DM m^−2^) of tall fescue (*Festuca arundinacea* Schreb.) and cocksfoot (*Dactylis glomerata* L.).

	***F. arundinacea***				***D. glomerata***			
	2017		2018		2017		2018	
	1st cut	2nd cut	1st cut	2nd cut	1st cut	2nd cut	1st cut	2nd cut
CTR	0.000 c	0.000 c	0.001 b	0.011 c	0.028 b	0.027 b	0.003 a	0.047 a
Z	0.073 abc	0.147 b	0.030 ab	0.109 a	0.176 a	0.291 a	0.021 a	0.063 a
B350	0.014 bc	0.000 c	0.001 b	0.019 bc	0.000 b	0.000 b	0.003 a	0.062 a
B350 + Z	0.090 ab	0.136 b	0.032 ab	0.092 a	0.148 a	0.290 a	0.015 a	0.054 a
B550	0.021 bc	0.000 c	0.010 b	0.065 ab	0.000 b	0.016 b	0.004 a	0.053 a
B550 + Z	0.141 a	0.258 a	0.052 a	0.089 a	0.158 a	0.273 a	0.015 a	0.067 a

For each column, mean values with different letters are significantly different (*P* < 0.05, n = 3); Bonferroni post hoc test.

The soil amendments used in this research significantly affected grass productivity. The lowest yield of tall fescue was obtained in the contaminated soil without amendments, CTR (annually 0.0057 kg DM m^−2^). All amendments used in this study significantly increased tall fescue productivity. B350 resulted in annual yields that were 3 times higher than those under CTR, and even better results were observed when B550 was applied (0.485 kg DM m^−2^ annually). However, when zeolite was used, the biomass production of tall fescue significantly increased in comparison with that under CTR and biochars B350 and B550. This effect was observed during all harvests in 2017 and 2018.

Cocksfoot produced significantly more biomass under CTR than tall fescue (0.0523 kg DM m^-2^ annually). When biochar B350 was used as a soil amendment, cocksfoot did not produce any aboveground biomass during the 1st and 2nd cuts in 2017. A similar effect was also observed for biochar B550. These two amendments resulted in lower cocksfoot yield than that under CTR. Much higher biomass production was observed in the treatments with zeolite either alone or in mixture with biochar B350 or B550. This effect was particularly notable in 2017. In the second year, the differences among treatments disappeared, and in the 1st and 2nd cut, the biomass was at the same level in all treatments.

### Root biomass and morphometric parameters

**Figure 3:**
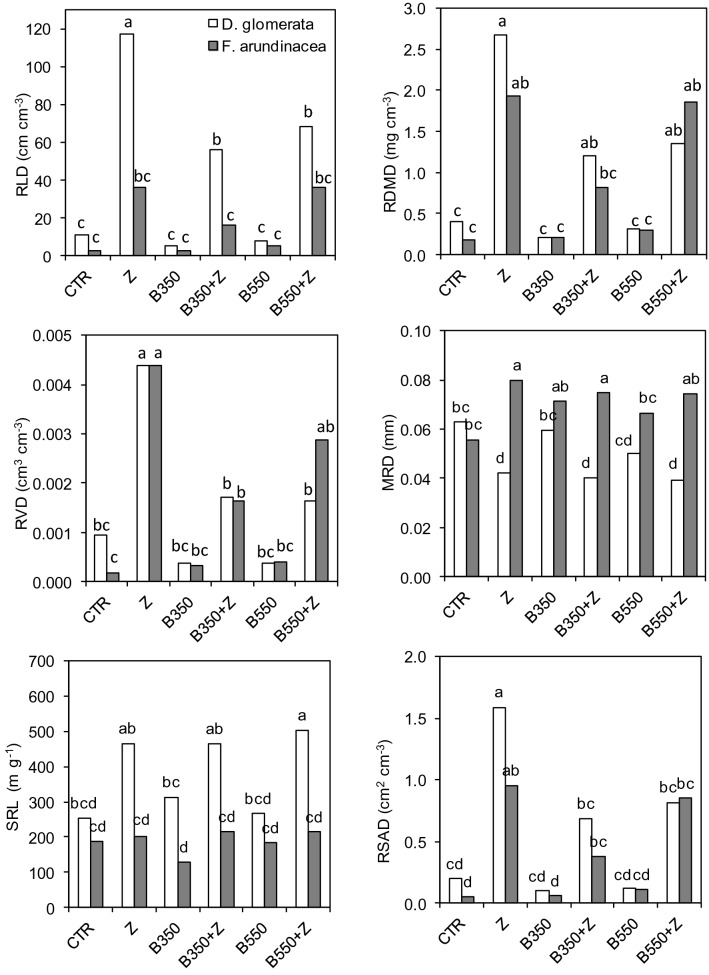
Root morphometric characteristics for soil amendments treatment. Treatments: zeolite (Z), biochars (B350 and B550), mixtures of biochars and zeolite (B350 + Z and B550 + Z), control (CTR). Different letters on the bars indicate significant differences by the Bonferroni test (*P* < 0.05, n = 6).

The root system of cocksfoot was characterized by a slightly higher mass (RDMD = 1.03 mg cm^−3^ on average) than that of tall fescue (0.88 mg cm^−3^) (Fig. 3). However, the roots of cocksfoot grew close together in bunches, which resulted in a very high RLD (44.3 cm cm^−3^); the RLD for tall fescue was only 16.36 cm cm^−3^. This difference in root system morphology also resulted in a higher root diameter (MRD) in tall fescue (0.070 mm) than in cocksfoot (0.049 mm).

The soil amendments zeolite and biochar used in the contaminated soil significantly affected the root biomass (Fig. 3). Tall fescue produced the lowest root biomass under CTR, without any soil amendments (RDMD = 0.17 mg cm^−3^). When biochars B350 and B550 were added, the RDMD increased to 0.21 and 0.30 mg cm^-3^, respectively. However, these values were not significantly different from that under CTR (*P* > 0.05). The highest results were obtained when zeolite was applied. Zeolite with biochar B350 resulted in a root dry matter of 0.82 mg cm^−3^, and in mixture with B550, the root dry matter increased to 1.86 mg cm^−3^. However, the highest value of RDMD was observed when zeolite alone, without biochar, was used (1.93 mg cm^−3^). The cocksfoot root biomass under CTR resulted in a higher RDMD (0.40 mg cm^-3^) than that under the biochar treatments (0.26 mg cm^-3^ on average). As in tall fescue, zeolite application resulted in significantly higher root biomass in cocksfoot, with the highest RDMD in the Z treatment (2.68 mg cm^−3^).

**Figure 4:**
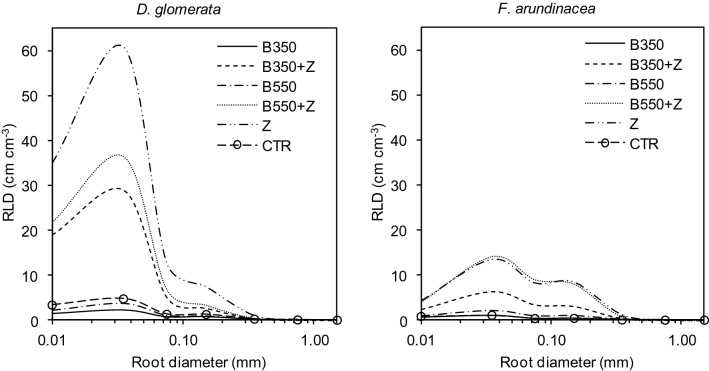
Root length density (RLD) distribution at the different root diameter values for treatments with different soil organic amendments. Treatments: zeolite (Z), biochars (B350 and B550), mixtures of biochars and zeolite (B350 + Z and B550 + Z), control (CTR).

The results for root length correspond to the relationships observed in RDMD. Tall fescue produced more roots when zeolite was added to the contaminated soil (Fig. 4). However, the RLD values for all treatments were not significantly different. The highest RLD for cocksfoot was observed in the treatment with zeolite (117 cm cm^−3^). This value was two times higher than those under B350 + Z and B550 + Z. When biochar without zeolite was used, the RLD reached 5.23 and 7.80 cm cm^−3^ in the B350 and B550 treatments, respectively. Similar relationships were also noted for the RVD and RSAD parameters. The highest values of root volume and surface area were calculated for the treatment in which only zeolite was applied. Unlike their values for RDMD and RLD, both investigated grass species had similar mean values of RVD and RSAD. SRL, as a derivative of RDMD and RLD, was significantly higher in all the treatments with zeolite.

Tall fescue and cocksfoot differed with regard to root diameter. The roots of tall fescue were significantly thicker (MRD = 0.073 mm) than those of cocksfoot (0.049 mm). These two grasses presented different responses to the soil amendments applied to the toxic metal-contaminated soil. The highest value of MRD in cocksfoot was observed under CTR (0.063 mm). The application of the biochars slightly reduced the root diameter. However, significantly lower MRD values were obtained in all treatments in which zeolite was applied, whether alone or in mixture with biochar. The root diameter of tall fescue was similar in all treatments (0.075 mm) except CTR and B550, in which slightly thinner roots were observed (0.056 and 0.067 mm, respectively).

**Figure 5:**
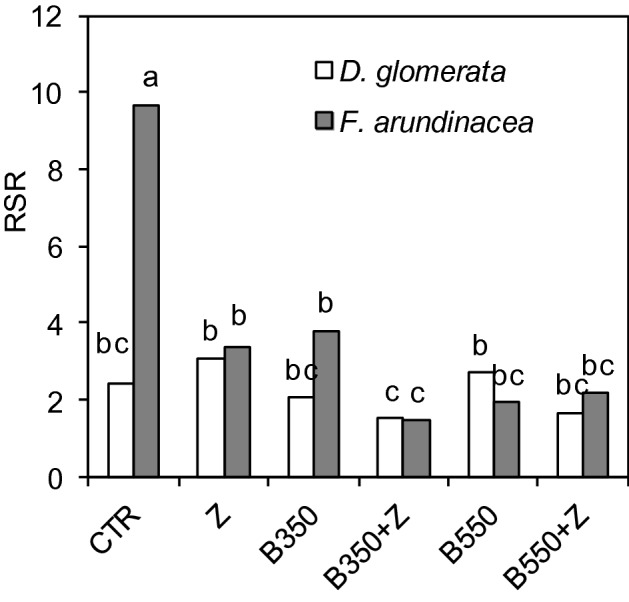
Root:shoot ratio (RSR) for two grass species on soil remediated with biochar and zeolite. Treatments: zeolite (Z), biochars (B350 and B550), mixtures of biochars and zeolite (B350 + Z and B550 + Z), control (CTR). Different letters on the bars indicate significant differences by the Bonferroni test (*P* < 0.05, n = 6).

**Figure 6:**
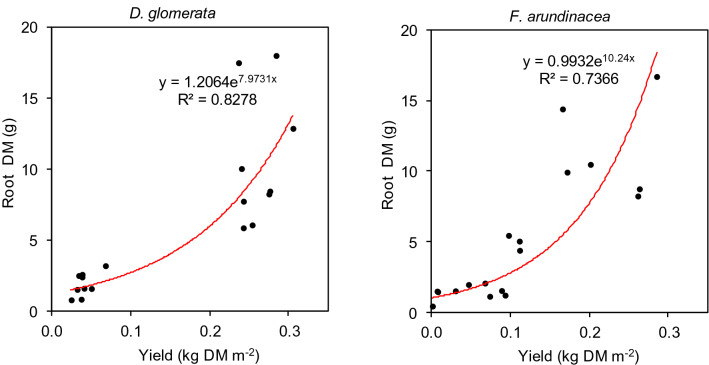
Relationship between root dry matter and annual aboveground biomass productivity of tall fescue and cocksfoot. Solid line is the fitted non-linear regression.

The RSR parameter of cocksfoot did not change in response to the soil amendments used in this research (2.99 on average). However, some differences appeared in tall fescue. The highest RSR value was calculated under CTR (9.67), and the lowest was calculated under B350 + Z (1.48) (Fig. 5). In addition, the correlation between aboveground biomass and root biomass was statistically significant (correlation coefficient k = 0.83, *P* < 0.05). Regression models for the root:shoot ratios are presented in Fig. 6. These relationships in both grass species were not linear. They were best represented as exponential models.

## Discussion

The present investigation has shown the beneficial effect of zeolite when used as a soil amendment in toxic metal-contaminated soil. This effect was shown on perennial grass species, which are widely used in phytoremediation. The biochar amendments did not improve cocksfoot productivity and only slightly affected tall fescue productivity in contaminated soil. Studies have reported that in conventional crop production on non-contaminated soil, biochar is recognized as a soil amendment that significantly improves plant productivity^28^. For some crops, e.g., maize, this effect is more pronounced in the second and third years after biochar application. Fiaz et al.^29^, in research on toxic metal remediation through biochar application, showed significant improvement in *Brassica campestris* L. growth as a result of lower metal uptake. Biochar reduced the availability of heavy metals due to its metal sorption ability caused by its structural modification during pyrolysis. The effect of biochar application on plant growth was particularly noticeable in combination with mineral fertilization. Rehman et al.^30^ reported that biochar enhanced rice biomass because of its Cd immobilization potential and its function as a source of plant nutrients. A similar effect was reported by Xu et al.^31^ in an experiment with maize on Cd-contaminated soil remediated with biochar. Rehman et al.^32^, in a field trial with maize, showed that biochar increased pH and decreased bioavailable Ni in the soil. The beneficial effect of biochar on soil remediation is more pronounced in annual than in perennial crops. In this study, this effect was not confirmed for cocksfoot.

In the present research, zeolite application resulted in significantly higher biomass production in tall fescue and cocksfoot compared with that under the control and biochar-amended treatments. Butorac et al.^33^ reported the positive fertilizing effect of zeolite on the yields of winter wheat, maize, soybean and winter barley. This effect was also observed when zeolites were used in the remediation of soil contaminated with toxic metals. Wang et al.^34^ reported that in a mixture with compost, zeolite was shown to promote plant growth in contaminated soil.

Biochar and zeolite improve crop productivity during the remediation process through various mechanisms, i.e., toxic metal adsorption, increased soil water retention, supporting plant growth-promoting microorganisms, and increased root development. In a previous study, Głąb et al.^17^ reported that biochar improved the physical properties of sandy soil, e.g., its soil porosity and water retention characteristics. Zeolite was also recognized to decrease bulk density and increase total porosity, which results in higher soil water content. Zeolite application increases the soil water use efficiency through higher hydraulic conductivity and water retention^35^. The amendment of sand with zeolite can increase the water available to plants by 50%^36^. Zeolite is one of the most efficient cationic exchangers and has a high capacity for toxic metal adsorption^10^. Its cationic interchange capacity is greater than that of other soil minerals. Thus, zeolites are widely used as soil amendments to increase nutrient retention capacity^37^.

In this study, zeolite and biochar influenced the root biomass and root morphometric parameters. However, zeolite affected the root system of the investigated grass species to a greater extent than biochar. This is not in line with other studies, where biochar was recognized as an effective amendment that favoured root growth as a result of chemical and biological processes. Rees et al.^38^ attributed root growth changes in the presence of biochar to decreases in both soil phytotoxicity and nutrient availability. Bioavailable metal concentrations decrease with increasing concentrations of biochar amendments. Houben et al.^39^ reported that biochar in soil reduced metal concentrations in shoots and tripled the biomass production of *Brassica napus* L. as a result of the soil fertility improvement. Reibe et al.^40^ suggested that nutrients released from biochar affect the root morphology of spring wheat. However, this effect depends on the feedstock, the production process and the amount of biochar used.

Zeolite was also reported to be an amendment that positively affects root growth in contaminated soils. Lahori et al.^10^ confirmed this effect for cabbage and maize in multiple contaminated soils remediated with zeolite. Shahbaz et al.^41^ showed that zeolite and biochar increased sunflower and maize growth and improved their root system morphology in soil contaminated with Ni. They also observed an interaction effect between these two soil amendments. The current study confirms the positive effect of zeolite on grass roots. However, an interaction effect of the zeolite and biochar combination was not observed. The lack of an interaction effect may be due to the type of species used in this study, namely, perennial grass species.

Xiang et al.^42^, in their meta-analysis, stated that biochar amendment improved root characteristics such as RDM, RVD and RSA. The biochar influenced to a greater extent on RLD and number of root tips more than the RDM. This suggests that biochar used as soil amendment has a beneficial effect on root morphology to reduce plant nutrient and water deficiencies rather than maximize biomass accumulation. A similar effect was also observed by Olmo et al.^43^. High rates of biochar increased the SRL and decreased MRD and root tissue mass density, what indicate better development of fine root. This improved nutrients and water acquisition by increasing biochar-root interactions, soil penetration and fertilizer efficiency.

The abovementioned relationship can be confirmed by the root:shoot ratio. In this study, RSR was significantly higher in the control than in the treatments with biochar and zeolite, particularly for tall fescue. Plants allocate their biomass to the organ that is the most resource-limited^44^. Usually, the this optimization is expressed as a change in the biomass allocations to the shoots and roots in response to nutrient availability^45,46^. In soil contaminated with toxic metals, this optimization can be related to the bioavailability of metals.

Root morphometric parameter could be used as predictors of susceptibility to metal contaminated soil and usefulness in phytoremediation. Long‐term survivors of grasses in contaminated soils can be explained by two mechanisms: (1) dense and deep root system which can reach soil layer with lower metal content and (2) ability to reduced metal uptake^47^. This study show that in metal contaminated soil cocksfoot produce longer root than tall fescue. However, tall fescue roots were thicker than these of cocksfoot. The relationship between root characteristics of grasses and vulnerability to contaminated soils needs further investigation.

## Conclusions

Our investigation shows that zeolite was a more effective soil amendment than biochar in the soil remediation process. No interaction between zeolite and biochar was observed. The temperature of the pyrolysis process was also found to be an insignificant parameter in terms of the biological results of the soil remediation.

Higher biomass production was observed in tall fescue and cocksfoot when zeolite was applied either alone or in mixture with biochars. Zeolite used in the contaminated soil significantly affected root biomass and root morphology parameters. Tall fescue and cocksfoot produced more roots when zeolite was used. Biochar as a soil amendment did not affect most root morphometric parameters. The application of biochars only slightly reduced the root diameter in cocksfoot. The root:shoot ratio was significantly higher in the control than in the treatments with biochar and zeolite. This effect can be ascribed to the optimization of biomass allocation by plants during environmental stress. It can be concluded that cocksfoot is a grass species that has greater resistance to toxic metal contamination than tall fescue.

## References

[1] Li ZY, Ma ZW, van der Kuijp TJ, Yuan ZW, Huang L (2014). A review of soil heavy metal pollution from mines in China: pollution and health risk assessment. Sci. Total Environ..

[2] Zhou C, Yuan H, Ning C, Li S, Xia Z, Zhu M, Ma Q, Yu W (2020). Evaluation of different types and amounts of amendments on soil Cd immobilization and its uptake to wheat. Environ. Manag..

[3] He Z, Shentu J, Yang X, Baligar VC, Zhang T, Stoffella PJ (2015). Heavy metal contamination of soils: sources, indicators, and assessment. J. Environ. Indic..

[4] Rodríguez-Eugenio, N., McLaughlin, M., Pennock, D. Soil Pollution: A Hidden Reality. Rome, FAO (2018).

[5] Lin C-F, Lo S-S, Lin H-Y, Lee Y (1998). Stabilization of cadmium contaminated soils using synthesized zeolite. J. Hazard. Mater..

[6] Aransiola SA, Ijah UJJ, Abioye OP, Bala JD (2019). Microbial-aided phytoremediation of heavy metals contaminated soil: a review. Eur. J. Biol. Res..

[7] Porter SK, Scheckel KG, Impellitteri CA, Ryan JA (2004). Toxic metals in the environment: thermodynamic considerations for possible immobilization strategies for Pb, Cd, As and Hg. Crit. Rev. Environ. Sci. Technol..

[8] Contin M, Miho L, Pellegrini E, Gjoka F, Shkurta E (2019). Effects of natural zeolites on ryegrass growth and bioavailability of Cd, Ni, Pb, and Zn in an Albanian contaminated soil. J. Soils Sedim..

[9] Bashir S, Salam A, Rehman M, Khan S, Gulshan AB, Iqbal J, Shaaban M, Mehmood S, Zahra A, Hu H (2019). Effective role of biochar, zeolite and steel slag on leaching behavior of Cd and its fractionations in soil column study. Bull. Environ. Contam. Toxicol..

[10] Lahori AH, Mierzwa-Hersztek M, Demiraj E, Sajjad RU, Ali I, Shehnaz I, Aziz A, Zuberi MH, Pirzada AM, Hassan K, Zhang Z (2020). Direct and residual impacts of zeolite on the remediation of harmful elements in multiple contaminated soils using cabbage in rotation with corn. Chemosphere.

[11] Mahabadi AA, Hajabbasi MA, Khademi H, Kazemian H (2007). Soil cadmium stabilization using an Iranian natural zeolite. Geoderma.

[12] Yi N, Wu Y, Fan L, Hu S (2019). Remediating Cd-contaminated soils using natural and chitosan-introduced zeolite, bentonite, and activated carbon. Pol. J. Environ. Stud..

[13] Park JH, Choppala GK, Bolan NS, Chung JW, Chuasavathi T (2011). Biochar reduces the bioavailability and phytotoxicity of heavy metals. Plant Soil.

[14] Atkinson CJ, Fitzgerald JD, Hipps NA (2010). Potential mechanisms for achieving agricultural benefits from biochar application to temperature soils: a review. Plant Soil.

[15] Peake LR, Reid GJ, Tang X (2014). Quantifying the influence of biochar on the physical and hydrological properties of dissimilar soils. Geoderma.

[16] Mukherjee A, Lal R (2013). Biochar impacts on soil physical properties and greenhouse gas emissions. Agronomy.

[17] Głąb T, Palmowska J, Zaleski T, Gondek K (2016). Effect of biochar application on soil hydrological properties and physical quality of sandy soil. Geoderma.

[18] Li H, Dong X, da Silva EB, Oliveira L, Chen Y, Ma LQ (2017). Mechanisms of metal sorption by biochars: biochar characteristics and modifications. Chemosphere.

[19] Jadia CD, Fuleka MH (2008). Phytotoxicity and remediation of heavy metals by fibrous root grass (sorghum). J. Appl. Biosci..

[20] Bandura L, Franus M, Józefaciuk G, Franus W (2015). Synthetic zeolites from fly ash as effective mineral sorbents for land-based petroleum spills cleanup. Fuel.

[21] International Biochar Initiative. Standardized Product Definition and Product Testing Guidelines for Biochar That Is Used in Soil (aka IBI Biochar Standards), Version 2.0, IBI-STD-2.0 (2014).

[22] Gondek K, Mierzwa-Hersztek M (2016). Effect of low-temperature biochar derived from pig manure and poultry litter on mobile and organic matter-bound forms of Cu, Cd, Pb and Zn in sandy soil. Soil Use Manag..

[23] Brunauer S, Emmett PH, Teller E (1938). Adsorption of gases in multimolecular layers. J. Am. Chem. Soc..

[24] Barrett EP, Joyner LG, Halenda PP (1951). The determination of pore volume and area distributions in porous substances II. J. Am. Chem. Soc..

[25] Smucker AJM, McBurney SL, Srivastava AK (1982). Quantitative separation ofroots from compacted soil profiles by the hydropneumatic elutriation system. Agron. J..

[26] Bauhus J, Messier C (1999). Evaluation of fine root length and diametermeasurements obtained using RHIZO image analysis. Agron. J..

[27] Głąb T, Gondek K, Mierzwa-Hersztek M (2020). Pyrolysis improves the effect of straw amendment on the productivity of perennial ryegrass (*Lolium perenne* L.). Agronomy.

[28] Karthik A, Hussainy SAH, Rajasekar M (2020). Effect of biochar on the growth and yield of cotton and maize: a review. Int. J. Chem. Stud..

[29] Fiaz K, Danish S, Younis U, Malik SA, Raza Shah MH, Niaz S (2014). Drought impact on Pb/Cd toxicity remediated by biochar in *Brassica campestris*. J. Soil Sci. Plant Nutr..

[30] Rehman MZ, Batool Z, Ayub MA, Hussaini KM, Murtaza G, Usman M, Naeem A, Khalid H, Rizwan M, Ali S (2020). Effect of acidified biochar on bioaccumulation of cadmium (Cd) and rice growth in contaminated soil. Environ. Technol. Innov..

[31] Xu P, Sun CX, Ye XZ, Xiao WD, Zhang Q, Wang Q (2016). The effect of biochar and crop straws on heavy metal bioavailability and plant accumulation in a Cd and Pb polluted soil. Ecotoxicol. Environ. Saf..

[32] Rehman MZ, Rizwan M, Ali S, Fatima N, Yousaf B, Naeem A, Sabir M, Raza Ahmad H, Sik Ok Y (2016). Contrasting effects of biochar, compost and farm manure on alleviation of nickel toxicity in maize (*Zea mays* L.) in relation to plant growth, photosynthesis and metal uptake. Ecotoxicol. Environ. Saf..

[33] Butorac A, Filipan T, Basic F, Butorac J, Mesic M, Kisic I (2002). Crop response to the application of special natural amendments based on zeolite tuff. Rostlinná Výroba.

[34] Wang SB, Peng YL (2010). Natural zeolites as effective adsorbents in water and wastewater treatment. Chem. Eng. J..

[35] Nakhli SAA, Delkash M, Bakhshayesh BE, Kazemian H (2017). Application of zeolites for sustainable agriculture: a review on water and nutrient retention. Water Air Soil Pollut..

[36] Ozbahce A, Tari AF, Gönülal E, Simsekli N, Padem H (2015). The effect of zeolite applications on yield components and nutrient uptake of common bean under water stress. Arch. Agron. Soil Sci..

[37] De Smedt C, Someus E, Spanoghe P (2015). Potential and actual uses of zeolites in crop protection. Pest Manag. Sci..

[38] Rees F, Sterckeman T, Morel JL (2016). Root development of non-accumulating and hyperaccumulating plants in metal-contaminated soils amended with biochar. Chemosphere.

[39] Houben D, Evrard L, Sonnet P (2013). Beneficial effects of biochar application to contaminated soils on the bioavailability of Cd, Pb and Zn and the biomass production of rapeseed (*Brassica napus* L.). Biomass Bioenergy.

[40] Reibe K, Götz KP, Döring TF, Ros CL, Ellmer F (2015). Impact of hydro-/biochars on root morphology of spring wheat. Arch. Agron. Soil Sci..

[41] Shahbaz AK, Lewinska K, Iqbal J, Ali Q, ur Rahman M, Iqbal M, Abbas F, Tauqeer HM, Ramzani PMA (2018). Improvement in productivity, nutritional quality, and antioxidative defence mechanisms of sunflower (*Helianthus annuus* L.) and maize (*Zea mays* L.) in nickel contaminated soil amended with different biochar and zeolite ratios. J. Environ. Manag..

[42] Xiang Y, Deng Q, Duan H, Guo Y (2017). Effects of biochar application on root traits: a meta-analysis. GCB Bioenergy.

[43] Olmo M, Villar R, Salazar P, Alburquerque JA (2016). Changes in soil nutrient availability explain biochar’s impact on wheat root development. Plant Soil.

[44] McCarthy MC, Enquist BJ (2007). Consistency between an allometric approach and optimal partitioning theory in global patterns of plant biomass allocation. Funct. Ecol..

[45] Bonifas KD, Walters DT, Cassman KG, Lindquist JL (2005). Nitrogen supply affects root:shoot ratio in corn and velvetleaf (*Abutilon theophrasti*). Weed Sci..

[46] Agren GI, Franklin O (2003). Root:shoot ratios, optimization and nitrogen productivity. Ann. Bot..

[47] Palazzo AJ, Cary TJ, Hardy SE, Lee CR (2003). Root growth and metal uptake in four grasses grown on zinc-contaminated soils. J. Environ. Qual..

